# Prevalence of systemic lupus erythematosus in autoimmune hemolytic anemia patients based on coombs test results

**DOI:** 10.1186/s40001-025-02601-8

**Published:** 2025-05-02

**Authors:** Raja Iqbal Mulya Harahap, Tiara Ardiningrum, Yunisa Pamela, Ahmedz Widiasta, Rini Rossanti, Maulidwina Bethasari

**Affiliations:** 1 Department of Clinical Pathology, Faculty of Medicine, Universitas Padjadjaran/Hasan Sadikin Hospital, Bandung, West Java Indonesia; 2 Bachelor of Medicine, Faculty of Medicine, Universitas Padjadjaran, Bandung, West Java Indonesia; 3 Department of Basic Medical Science, Faculty of Medicine, Universitas Padjadjaran, Bandung, West Java Indonesia; 4 Department of Pediatrics, Faculty of Medicine, Universitas Padjadjaran/Hasan Sadikin Hospital, Bandung, Indonesia; 5 Department of Pharmacy, Faculty of Science and Technology, Universitas Muhammadiyah Bandung, Bandung, West Java Indonesia; 6 Department of Clinical Application and Research, Preanger Institute for Biomedical Engineering, Science, and Technology, Bandung, West Java Indonesia

**Keywords:** Coombs test, Autoimmune hemolytic anemia

## Abstract

**Background:**

Autoimmune hemolytic anemia (AIHA) is a rare blood disorder with an incidence of 1–3 per 100,000 people annually and a mortality rate of about 11%. AIHA is classified into warm, cold, and mixed types, which can be primary or secondary. Diagnosis is made through direct and indirect Coombs tests.

**Purpose:**

This study aims to describe Coombs test results in suspected AIHA patients at Dr. Hasan Sadikin General Hospital in Bandung from 2020 to 2022.

**Methods:**

A descriptive cross-sectional study was conducted with 78 AIHA cases. Blood samples were taken for both direct and indirect Coombs tests.

**Results:**

Among the 78 subjects, 83.33% were female, with an average age of 26.06 ± 15.91 years. The warm type of AIHA was most common (91.03%). Primary etiology was the most common in all AIHA types: 47.89% in warm, 66.67% in cold, and 50% in mixed. The most common direct Coombs test result was + 3 in warm (47.89%), cold (33.33%), and mixed (75%) AIHA. The most frequent indirect Coombs result was + 2 in warm (30.99%) and mixed (75%) AIHA, and + 3 in cold AIHA (66.67%).

**Conclusion:**

This study shows that AIHA at Dr. Hasan Sadikin General Hospital is more prevalent in females and younger individuals, with Systemic Lupus Erythematosus (SLE) being the most common secondary cause. Warm AIHA was the most frequent, with a rare occurrence of mixed AIHA.

**Supplementary Information:**

The online version contains supplementary material available at 10.1186/s40001-025-02601-8.

## Introduction

Autoimmune hemolytic anemia (AIHA) is a rare blood disorder characterized by autoantibodies binding to the surface membrane of erythrocytes, leading to premature destruction of red blood cells [1]. The incidence of AIHA is 1–3 per 100,000 per year, with a mortality rate of approximately 11%. AIHA is classified into warm AIHA (primarily caused by extravascular hemolysis mediated by warm-reactive IgG, accounting for 75% of all AIHA cases), cold AIHA (usually caused by intravascular hemolysis mediated by complement, accounting for about 15%), and mixed AIHA (less than 5%), based on the temperature range of autoantibodies involved in pathogenesis [1, 2]. Each type of AIHA can be further subclassified based on the presence or absence of underlying disease, categorized as primary or secondary [3, 4].

In some cases, AIHA may develop gradually, accompanied by physiological compensation [5]. Autoimmune hemolytic anemia can also present fulminantly with severe anemia that can be life-threatening. Specifically, warm IgM AIHA often experiences more severe hemolysis and higher mortality (up to 22%) compared to patients with other types of AIHA. In some patients, the presence of antibodies directly attached to red blood cells can be detected using a direct antiglobulin test (DAT). A positive Coombs test (DAT) is a diagnostic hallmark of AIHA; however, this may be absent in some pediatric cases, with a frequency ranging from 6 to 23% [6].

Laboratory diagnosis of AIHA can be assessed with direct and indirect Coombs tests. Laboratory diagnosis of AIHA relies on the results of a positive direct antiglobulin test (DAT) with anti-IgG (usually in warm AIHA) and/or anti-C3d (usually in cold AIHA), as well as supporting laboratory findings of hemolysis, such as increased serum lactate dehydrogenase (LDH), reticulocytosis, and spherocytosis on peripheral blood smear [7]. However, it is important to understand that not all cases of AIHA show a positive DAT result, as DAT may yield false negatives (up to 10% of all AIHA cases) due to IgA autoantibodies, low-affinity IgG, or IgG bound to red blood cells below the detection threshold of the test [4, 8]. More detailed AIHA type diagnosis to confirm either flase negative or false positive is explained in the previous study [9].

Systemic Lupus Erythematosus (SLE) is an autoimmune condition that can be associated with Autoimmune Hemolytic Anemia (AIHA), with patients often presenting both conditions simultaneously. AIHA can occur when there is a positive Direct Antiglobulin Test (DAT) for IgG, IgG and C3, or C3 alone. This concept was first established by Gilliland, Leddy, and Vaughan in 1970 through a complement-fixing test sensitive to complement fixation [9]. Typically, the DAT involves using a polyspecific reagent containing both IgG and complement C3. In some rare instances, autoimmune hemolysis may be suspected even if the DAT result is negative. In such cases, quantitative DAT testing can help identify less common antibody subtypes, aside from IgG or C3. A positive antiglobulin test result, in the absence of other confounding factors, typically indicates hemolysis caused by antibodies directed against native red blood cells. However, there is limited research on Coombs test profiles in AIHA patients in Indonesia, particularly in Bandung. Consequently, this study aims to investigate the prevalence of SLE in patients diagnosed with AIHA, specifically focusing on those with positive Coombs test results. The research also seeks to gather descriptive data on Coombs test outcomes in suspected AIHA patients at Dr. Hasan Sadikin General Hospital, to better understand the potential relationship between AIHA and SLE in this population.

## Methods

This study is a descriptive study with a cross-sectional design, where observations were made on specified variables at a single point in time to provide a profile of Coombs test results in suspected Autoimmune Hemolytic Anemia (AIHA) patients at Dr. Hasan Sadikin General Hospital in Bandung. The study utilized a retrospective approach, relying on secondary data from patient medical records spanning the period from 2020 to 2022, which met the established inclusion and exclusion criteria. Human Ethics and Consent to Participate Declarations was issued by Research Ethics Committee Universitas Padjadjaran Bandung (Registration number: 2404010503).

The inclusion criteria for this study were medical records of AIHA patients at Dr. Hasan Sadikin (RSHS) General Hospital who underwent Coombs testing during the 2020–2022 period, and whose records included complete data corresponding to the study variables. The exclusion criteria were medical records that did not contain complete data corresponding to the Study variables. The study included a sample of 78 AIHA patients (13 males and 65 females) aged between 4 months and 81 years who were treated at Dr. Hasan Sadikin General Hospital. The researchers collected secondary data from the medical records of AIHA patients at the hospital during the years 2020–2022.

Diagnosis was made and recorded by clinicians based on established clinical pathways for each disease, alongside the results of relevant laboratory tests. At RSHS, each disease is diagnosed according to clinical guidelines and pathways specific to that condition. For Non-Hodgkin Lymphoma (NHL), the clinical pathway involves performing a lymph node biopsy to obtain tissue for histopathological examination, which is critical for confirming the presence of malignancy, following guidelines such as the NCCN (National Comprehensive Cancer Network) protocols for lymphoma diagnosis [10]. For pregnancy, the clinical pathway involves using either ultrasound imaging or a urinary beta-HCG test, in line with obstetric guidelines that recommend these tests for confirming pregnancy and assessing gestational age. For Systemic Lupus Erythematosus (SLE), the diagnosis follows the ACR-EULAR classification criteria, which provide a standardized set of clinical and laboratory parameters for diagnosing SLE [11]. This clinical pathway emphasizes the importance of specific serological markers and clinical features, guiding clinicians in their diagnosis. For tuberculosis, the clinical pathway involves acid-fast bacillus (AFB) staining of sputum samples, which follows the WHO (World Health Organization) guidelines for TB diagnosis, emphasizing direct microscopic examination to detect the presence of Mycobacterium tuberculosis [12]. For thalassemia, the clinical pathway relies on hemoglobin electrophoresis to identify abnormal hemoglobin patterns, as recommended by hematology guidelines for diagnosing thalassemia and other hemoglobinopathies [13]. For hepatocellular carcinoma (HCC), the clinical pathway includes ultrasound (USG) imaging along with liver function tests, in accordance with the guidelines set forth by liver disease associations, such as the American Association for the Study of Liver Diseases (AASLD), which emphasize the importance of imaging and liver markers in the diagnosis of HCC, particularly in patients with chronic liver disease [14]. Finally, for viral hepatitis, rapid immunological tests are used to detect viral antigens or antibodies, following clinical guidelines for hepatitis diagnosis that recommend such tests to quickly identify and differentiate between various hepatitis viruses [14]. These laboratory tests, in conjunction with clinical pathways and guidelines, ensure accurate diagnosis and appropriate management for each condition.

We conducted the direct Coombs test to detect antibodies or complement proteins that might be bound to the surface of red blood cells. The procedure involved incubating the patient's red blood cells with antihuman globulin (Coombs reagent), which binds to any immunoglobulins (IgG) or complement components present. Agglutination, or clumping of the red blood cells, signaled a positive result, confirming the presence of these antibodies or complement proteins.

To differentiate between warm and cold types of AIHA, we varied the temperature conditions during the Coombs test. For each sample, we performed the test at two different temperatures. First, we tested at 37 °C, which is the body temperature, to evaluate IgG-mediated hemolysis typical of warm AIHA. The direct Coombs test at this temperature helped identify IgG antibodies and any complement components bound to the red blood cell surface. Second, we performed the test at 4 °C to assess the presence of cold agglutinins, which are IgM antibodies typically involved in cold AIHA. Testing at this temperature was crucial in identifying cold AIHA, where IgM antibodies mediate hemolysis at lower temperatures.

**Fig. 1:**
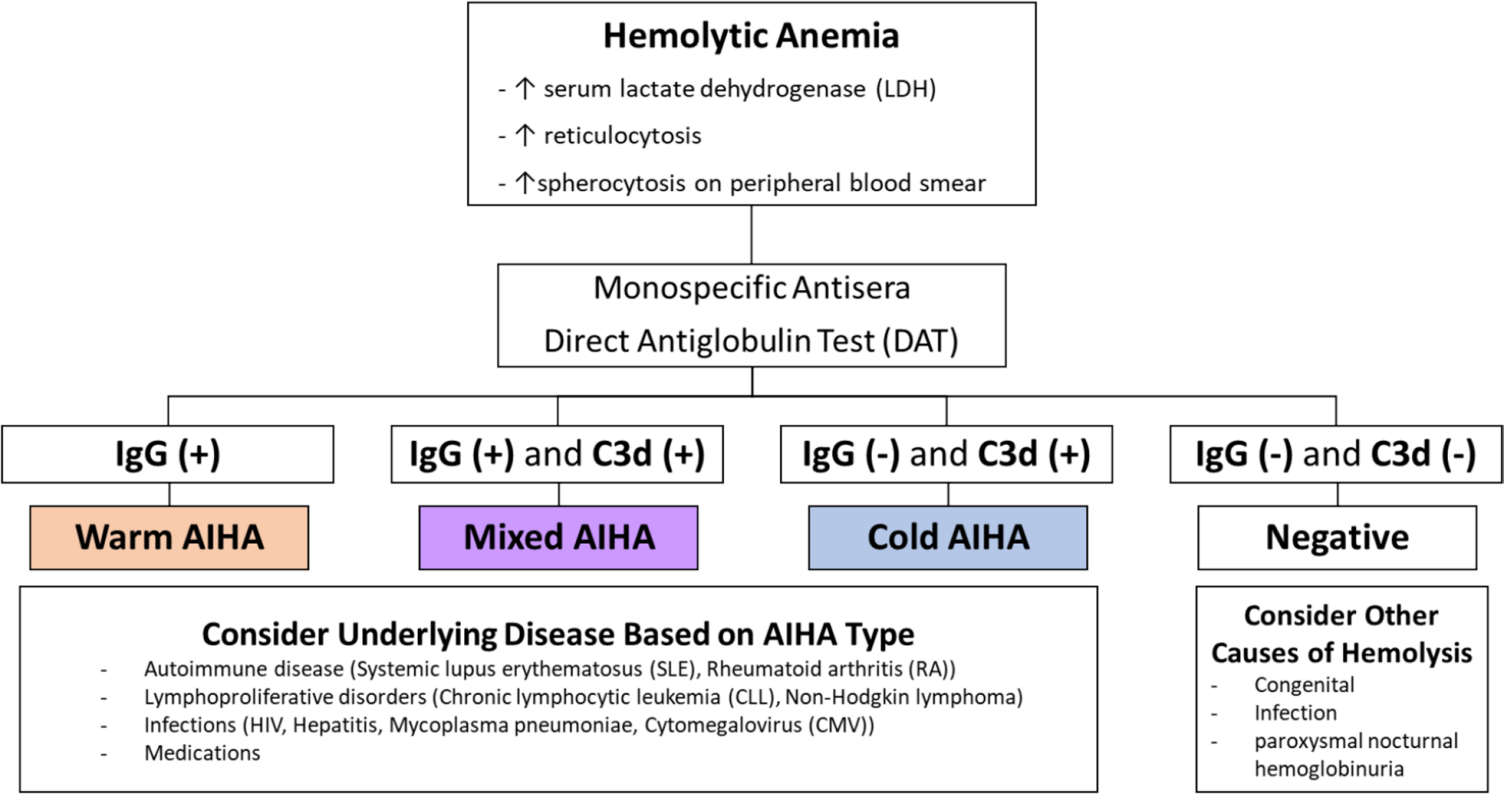
AIHA type diagnosis algorithm

We diagnosed warm AIHA when the direct Coombs test at 37 °C was positive for IgG antibodies, with or without the presence of complement (C3d). The test at 4 °C remained negative for cold agglutinins. For cold AIHA, we identified complement activation (C3d) in the test performed at 4 °C, without the detection of IgG antibodies at 37 °C. In cases of mixed AIHA, the Coombs test was positive at both 37 °C and 4 °C, indicating the presence of both IgG and IgM antibodies. The algorithm of the AIHA type diagnosis conducted is illustrated in Fig. 1.

## Result

The results of the study indicate that among the 78 subjects included, the majority were female (83.33%), with an average age of 26.06 ± 15.91 years. The age group of 17–25 years constituted the largest population with AIHA, accounting for approximately 33.33% of the cases. The subjects comprised 13 males (16.67%) and 65 females (83.33%). The most common type of AIHA among the subjects in this study was the warm type, representing 91.03% of the cases. The details are presented in Table 1 below.

**Tabel 1:** Research subjects characteristic

Characteristic	n (%)	Mean ± SD
Sex		
Male	13 (16.67)	
Female	65 (83.33)	
Age (Years)		26.06 ± 15.91
≤ 16	21 (26.92)	
17–25	26 (33.33)	
26–35	12 (15.38)	
36–45	8 (10.26)	
46–55	5 (6.41)	
56–65	4 (5.13)	
> 65	2 (2.56)	
Type		
Warm	71 (91.03)	
Cold	3 (3.85)	
Mix	4 (5.13)	

As shown in Table 2 below, primary etiology was the most common cause of AIHA among patients at Dr. Hasan Sadikin General Hospital in Bandung from 2020 to 2022, across all types of AIHA: warm, cold, and mixed, with percentages of approximately 47.89%, 66.67%, and 50%, respectively. Meanwhile, Systemic Lupus Erythematosus (SLE) was the most frequent secondary etiology among AIHA patients, with a prevalence of around 40.85% in warm AIHA and 50% in mixed AIHA.

**Tabel 2:** Distribution of AIHA Patients by Etiology, Direct Coombs Test Results, and Indirect Coombs Test Results at Dr. Hasan Sadikin General Hospital (2020–2022)

Characteristic etiology	Warm AIHA n (%)	Cold AIHA n (%)	Mix AIHA n(%)
Unclear	2 (2.82)		
Primary	34 (47.89)	2 (66.67)	2 (50)
Secondary			
NHL	1 (1.41)	1 (33.33)	–
Pregnancy	1 (1.41)	–	–
SLE	29 (40.85)	–	2 (50)
Tuberculosis	1 (1.41)	–	–
Thalasemia	1 (1.41)	–	–
HCC	1 (1.41)	–	–
Hepatitis viral	1 (1.41)	–	–
Direct coombs test			
Mixed field	1 (1.41)	1 (33.33)	
1	7 (9.86)		
2	7 (9.86)		
3	34 (47.89)	1 (33.33)	3 (75)
4	22 (30.99)	1 (33.33)	1 (25)
Indirect coombs test			
Negatif	21 (29.58)		
1	18 (25.35)		
2	22 (30.99)	1 (33.33)	3 (75)
3	10 (14.08)	2 (66.67)	1 (25)

^*^NHL, Non-Hodgkin Lymphoma; SLE, Systemic Lupus Erythematosus; TB, Tuberkulosis; HCC, Hepatocellular Carcinoma

**Fig. 2:**
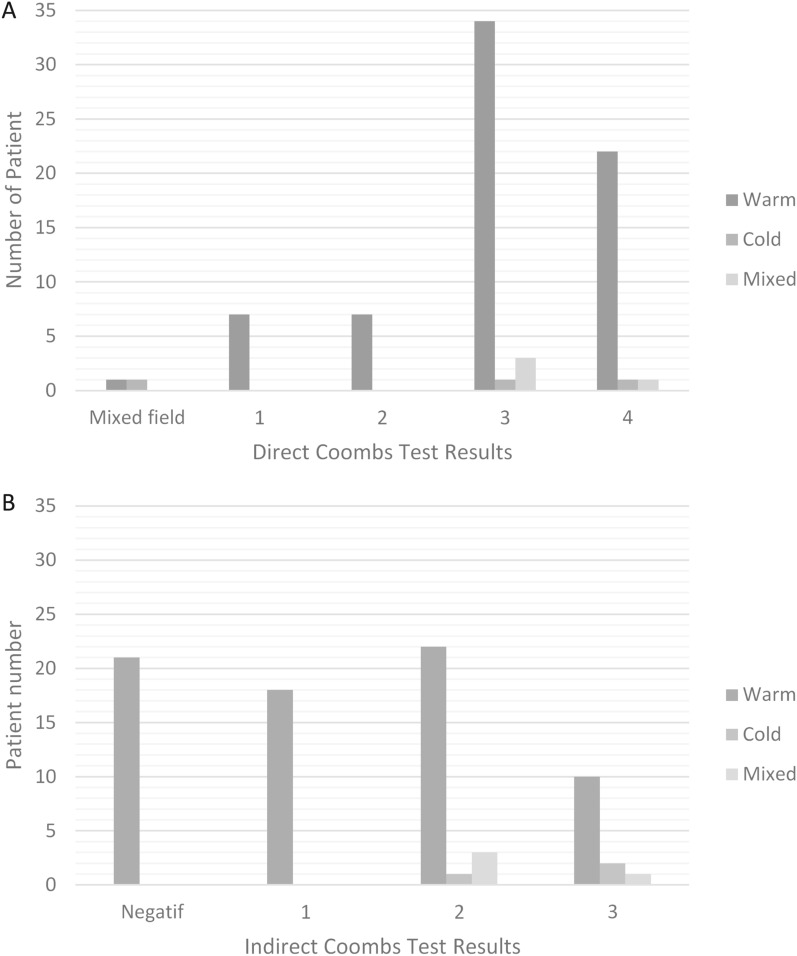
The comparison of Direct (**A**) and Indirect (**B**) Coombs test

In Fig. 2, it is illustrated that the most common result of the direct Coombs test was a + 3 in patients with warm, cold, and mixed AIHA, with percentages of approximately 47.89%, 33.33%, and 75%, respectively. For the indirect Coombs test, the most frequent result was + 2 in patients with warm AIHA (30.99%) and mixed AIHA (75%), and + 3 in patients with cold AIHA (66.67%).

## Discussion

Autoimmune hemolytic anemia (AIHA) is an acquired condition characterized by hemolysis that becomes decompensated due to the body's immune system attacking its own erythrocyte antigens [5]. The general diagnostic approach to AIHA primarily involves confirming the presence of anemia caused by hemolysis and obtaining serological evidence of anti-erythrocyte antibodies, which can be detected through the direct Coombs test or direct antiglobulin test (DAT) [4, 6]. Additionally, the indirect Coombs test or indirect antiglobulin test (IAT) can be employed to detect autoantibodies present in the serum. Circulating immunoglobulins in the serum will bind to reagent cells and can be detected by antiglobulin sera, which is indicated by the occurrence of agglutination (clumping) [4, 15, 16]. This diagnostic framework is crucial for confirming AIHA and understanding the underlying immunological mechanisms leading to erythrocyte destruction. The presence of autoantibodies and the subsequent positive Coombs test results provide essential insights into the pathogenesis and allow for appropriate clinical management of the condition [1, 17].

In this study, the majority of subjects were female (83.33%), with an average age of 26.06 ± 15.91 years, and the largest age group with AIHA was 17–25 years, accounting for approximately 33.33% of the cases. These findings are consistent with study by Fattizo et al., which found the estimated incidence of AIHA was 0.8–3 out of 100 000 individuals per year and is predominant in females [9]. Similarly, other study by Habiba et al. found that AIHA mostly found in women, compring 71% of the population included in the study [18]. The gender predilection observed in AIHA can be attributed to the higher prevalence of autoimmune diseases in women, which is potentially linked to the presence of two X chromosomes and the influence of sex hormones like estrogen. These factors are thought to play a role in the immune system's regulation, possibly contributing to the increased susceptibility of women to autoimmune conditions such as AIHA [19, 20].

In this study, AIHA was frequently found in younger individuals. This may be due to the high prevalence of primary etiology and secondary etiology related to Systemic Lupus Erythematosus (SLE) observed in this study [21]. This finding aligns with the literature, which states that autoimmune hemolytic anemia can occur at any age, with primary AIHA being the most common type among younger individuals [22]. Secondary etiologies, such as autoimmune diseases—particularly SLE—also predominantly affect young women [19, 20]. However, the risk of AIHA increases with age, with the risk of warm AIHA being five times higher in individuals in their 70s compared to those in their 40s [21, 23].

The primary reason AIHA tends to affect older adults could be due to the gradual decline in immune system function associated with aging (immunosenescence) or the accumulation of epigenetic abnormalities in hematopoietic cells over time. Aging processes, along with various comorbidities, increase the likelihood and severity of oxidative stress and eryptosis—a process involving changes in the erythrocyte membrane that leads to premature aging and death of red blood cells [24]. Genetic background, immunodeficiency, autoimmune diseases, infections, medications (particularly anticancer drugs), neoplasia (especially chronic lymphocytic leukemia, CLL), and transplantation have all been identified as significant risk factors for the development of AIHA, regardless of age. These factors contribute to the complex pathogenesis of AIHA and highlight the importance of considering a wide range of potential etiologies when diagnosing and managing this condition [25].

In this study, the most common type of AIHA among the subjects was the warm type, accounting for 91.03% of cases. This finding is consistent with the study by Rajabto et al., which reported the incidence of warm, cold, and mixed AIHA as 92%, 6%, and 2%, respectively, indicating that the majority of AIHA cases in that study were also warm AIHA (92%) [26]. Similarly, the study by Sulaiman et al. found that the majority of AIHA cases were of the warm type, representing 40.8% of cases [27].

Cold AIHA is indeed less commonly encountered compared to warm AIHA, accounting for only 10–20% of AIHA cases [23]. According to the literature, the current estimated incidence of AIHA is 1.77 cases per 100,000 per year, with warm AIHA being the most common type, comprising approximately two-thirds of all cases. The type of antibody involved in AIHA is identified using monospecific antibodies against immunoglobulin G (IgG) and C3d. When red blood cells are coated with IgG or IgG plus C3d, the antibodies are typically warm antibodies. In contrast, when red blood cells are coated only with C3d, the antibodies are often, but not always, cold antibodies [1, 2].

In this study, primary etiology was the most common cause among AIHA patients at Dr. Hasan Sadikin General Hospital from 2020 to 2022, across all types of AIHA: warm, cold, and mixed, with percentages of approximately 47.89%, 66.67%, and 50%, respectively. Systemic Lupus Erythematosus (SLE) was the most frequent secondary etiology among AIHA patients, with 40.85% in warm AIHA and 50% in mixed AIHA. According to the study by Sudulagunta et al., the most common causes of secondary warm AIHA include B-cell lymphoma, SLE, rheumatoid arthritis, chronic lymphocytic leukemia (CLL), immunodeficiency, renal cell carcinoma, and the use of certain medications (such as methyldopa and carbamazepine) [7].

These results are also in line with the study by Naithani, which reported that out of 79 AIHA patients, 27 (34%) had secondary AIHA [28]. Another study by Rajabto et al. found that the etiology of warm AIHA in their study was primarily divided into primary (54.3%) and secondary (45.7%) causes, with the majority of secondary AIHA cases being attributed to SLE (41.3%), followed by autoimmune hepatitis (2.2%) and chronic lymphocytic leukemia (2.2%) [26]. Environmental and genetic factors may influence the variability in SLE prevalence between different populations, which could explain the differences observed in this study compared to others. SLE is more frequently found in Asian populations compared to Caucasians [29].

In this study, we found that 40% of autoimmune hemolytic anemia (AIHA) cases were secondary to systemic lupus erythematosus (SLE), highlighting the high prevalence of SLE as a major cause of AIHA. This emphasizes the importance of diagnosing hemolytic anemia in SLE patients, particularly given its potential to affect disease management and patient outcomes. AIHA can manifest at any point in the course of SLE, even preceding the diagnosis of SLE itself in some cases. It is crucial to identify hemolytic anemia early because its delayed diagnosis or inappropriate management can lead to severe complications, including organ damage from anemia-induced hypoxia or the exacerbation of SLE symptoms. In addition, AIHA is often associated with thromboembolism, and patients with lupus anticoagulant (LAC) or anticardiolipin (ACL) antibodies who have an underlying AIHA background are at a heightened risk of thrombosis. This underscores the need for clinicians to be vigilant in monitoring these patients for both hematological and thrombotic complications. This complex interplay of factors underlines the importance of understanding the diverse etiologies and demographics associated with AIHA in different populations, which is crucial for tailoring appropriate diagnostic and therapeutic strategies.

Our study also observed that, among the 31 SLE-related AIHA cases, 2 patients exhibited mixed AIHA, a rare manifestation. Warm AIHA is the most common type (60–70% of cases), followed by cold AIHA (20–25%), and mixed AIHA, which is the rarest, accounting for about 5–10% of AIHA cases [5]. A study by Mayer et al. found that among 2,194 patients with detected warm autoantibodies, only 2 patients (less than 0.1%) displayed features consistent with mixed warm/cold AIHA, highlighting the rarity of this condition [30].

In SLE, the anti-erythrocyte antibodies are predominantly warm IgG, although mixed AIHA has also been reported [31, 32, 32]. Cold agglutinin AIHA can be either primary (cold agglutinin disease) or secondary to other conditions, including infections, malignancies, or autoimmune disorders [31]. Cold AIHA involves the activation of the classical complement pathway upon antibody binding to red blood cells, leading to the formation of the membrane attack complex and intravascular hemolysis. If the classical pathway fails, erythrocytes are opsonized with complement proteins, promoting phagocytosis in the liver and spleen and resulting in extravascular hemolysis [33]. This distinction between intravascular and extravascular hemolysis can influence treatment decisions, as corticosteroids, rituximab, IV immunoglobulin, and splenectomy are potential treatment options for AIHA [32].

Mixed AIHA presents a complex diagnostic challenge, and optimal management strategies are not well established [30]. In one case, the patient was untreated for an extended period before admission, which likely led to the delayed appearance of combined SLE and AIHA symptoms [34, 35]. Previous case reports, such as the one by Socol et al., have shown that mixed-type AIHA is frequently associated with SLE and lymphoma [36, 37]. While mixed-type AIHA tends to be less clinically aggressive than cold agglutinin disease, it is often more severe and chronic due to the hemolytic anemia it causes [37]. Although mixed-type AIHA often initially responds well to steroid therapy, it remains a chronic disease and patients often require multiple lines of therapy before achieving adequate control [17, 30]. Long-term remission after tapering or discontinuing steroids is uncommon, especially in severely affected patients [17, 30]. Consequently, patients may become steroid-dependent or require high doses for prolonged periods. The use of rituximab in managing mixed warm/cold AIHA remains an area of interest as two prospective randomized trials have explored the addition of rituximab upfront in patients with warm AIHA, but the response to steroid therapy has been mixed, and splenectomy has sometimes been necessary in cases of persistent hemolysis [17, 30]. On the other hand, some cases showing clinical improvement after treatment with both steroids and rituximab. A case of severe mixed-type AIHA, treated with both therapies, demonstrated stability in hemoglobin levels and improvement in hemolytic indices, highlighting the potential benefit of a combination approach [30]. However, variability in diagnostic criteria across sources complicates the diagnosis and management of this rare pathology. Hence, confirming the presence of cold agglutinins, which are active at temperatures ≥ 30 °C, is essential in patients with SLE and warm-type AIHA [30].

Due to the resources limitation, AIHA type diagnosis results was not confirmed further, which is one of the limitations of this study. The current study has lack of a detailed analysis of different antibody types, which limits a comprehensive understanding of the clinical course and pathogenesis of mixed-type AIHA. Furthermore, the treatment responses, particularly for rare cases of mixed-type AIHA, are not well documented. To address these gaps, further research is warranted, particularly focusing on the prevalence and role of various antibody types, as this could offer a deeper understanding of mixed-type AIHA. Additionally, studies examining the implications of vascular and extravascular hemolysis could provide valuable insights into the underlying mechanisms of the disease. Finally, there is a need for more research on effective treatment strategies for cold and mixed-type AIHA, with a focus on improving patient outcomes and management of these rare conditions.

## Conclusion

In conclusion, this study found a female predominance and a higher incidence in younger individuals, with Systemic Lupus Erythematosus (SLE) being the most common secondary cause of AIHA. Warm AIHA was the most prevalent type, while mixed AIHA was rare and challenging to manage. The study highlights the need for tailored diagnostic and therapeutic strategies to improve outcomes for AIHA patients.

## Supplementary Information

Supplementary Material 1.

Supplementary Material 2.

## Data Availability

No datasets were generated or analysed during the current study.
