# Supplementary material for: Prevalence of systemic lupus erythematosus in autoimmune hemolytic anemia patients based on coombs test results
Source: Eur J Med Res. 2025 May 2;30:351. doi: 10.1186/s40001-025-02601-8 (PMC12046922; doi:10.1186/s40001-025-02601-8)
Supplement: Supplementary file 1 — Supplementary Material 1. [file 40001_2025_2601_MOESM1_ESM.docx]

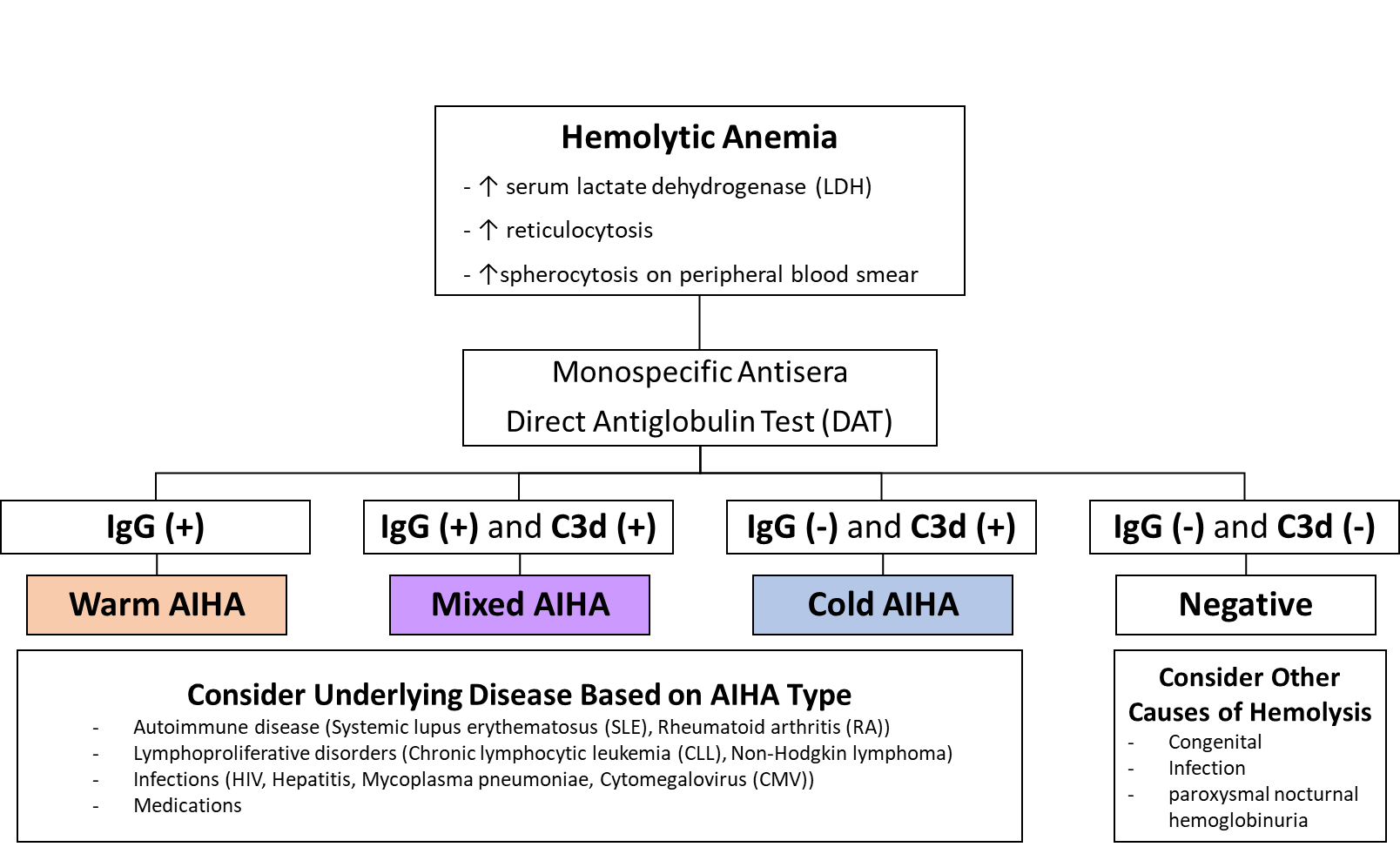


**Figure 1.** AIHA type diagnosis algorithm

A

B

**Figure 2.** The comparison of Direct (A) and Indirect (B) Coombs test
