# Supplementary material for: Prevalence of systemic lupus erythematosus in autoimmune hemolytic anemia patients based on coombs test results
Source: Eur J Med Res. 2025 May 2;30:351. doi: 10.1186/s40001-025-02601-8 (PMC12046922; doi:10.1186/s40001-025-02601-8)
Supplement: Supplementary file 2 — Supplementary Material 2. [file 40001_2025_2601_MOESM2_ESM.docx]

**Tabel 1.** Research Subjects Characteristic

| **Characteristic** | **n (%)** | **Mean ± SD** |
| --- | --- | --- |
| **Sex** |  |  |
| Male | 13 (16.67) |  |
| Female | 65 (83.33) |  |
| **Age (Years)** |  | 26.06 ± 15.91 |
| ≤ 16 | 21 (26.92) |  |
| 17 – 25 | 26 (33.33) |  |
| 26 – 35 | 12 (15.38) |  |
| 36 – 45 | 8 (10.26) |  |
| 46 – 55 | 5 (6.41) |  |
| 56 – 65 | 4 (5.13) |  |
| >65 | 2 (2.56) |  |
| **Type** |  |  |
| Warm | 71 (91.03) |  |
| Cold | 3 (3.85) |  |
| Mix | 4 (5.13) |  |

**Tabel 2.** Distribution of AIHA Patients by Etiology, Direct Coombs Test Results, and Indirect Coombs Test Results at Dr. Hasan Sadikin General Hospital (2020-2022)

| **Characteristic** | **Warm AIHA n (%)** | **Cold AIHA n (%)** | | **Mix AIHA n(%)** |
| --- | --- | --- | --- | --- |
| **Etiology** |  |  |  |  |
| **Unclear** | 2 (2.82) |  | |  |
| **Primary** | 34 (47.89) | 2 (66.67) | 2 (50) | |
| **Secondary:** |  |  |  | |
| NHL | 1 (1.41) | 1 (33.33) | - | |
| Pregnancy | 1 (1.41) | - | - | |
| SLE | 29 (40.85) | - | 2 (50) | |
| Tuberculosis | 1 (1.41) | - | - | |
| Thalasemia | 1 (1.41) | - | - | |
| HCC | 1 (1.41) | - | - | |
| Hepatitis viral | 1 (1.41) | - | - | |
| **Direct Coombs Test** |  |  |  | |
| **Mixed Field** | 1 (1.41) | 1 (33.33) |  | |
| **1** | 7 (9.86) |  |  | |
| **2** | 7 (9.86) |  |  | |
| **3** | 34 (47.89) | 1 (33.33) | 3 (75) | |
| **4** | 22 (30.99) | 1 (33.33) | 1 (25) | |
| **Indirect Coombs Test** |  |  |  | |
| **Negatif** | 21 (29.58) |  |  | |
| **1** | 18 (25.35) |  |  | |
| **2** | 22 (30.99) | 1 (33.33) | 3 (75) | |
| **3** | 10 (14.08) | 2 (66.67) | 1 (25) | |

*NHL, Non-Hodgkin Lymphoma; SLE, Systemic Lupus Erythematosus; TB, Tuberkulosis; HCC, Hepatocellular Carcinoma
